# Comparative Analysis of Carrier-Based Obturation and Lateral Compaction: A Retrospective Clinical Outcomes Study

**DOI:** 10.1155/2012/954675

**Published:** 2012-04-11

**Authors:** Robert Hale, Robert Gatti, Gerald N. Glickman, Lynne A. Opperman

**Affiliations:** ^1^Department of Endodontics, Texas A&M Health Science Center, Baylor College of Dentistry, 3302 Gaston Avenue, Dallas, TX 75246, USA; ^2^Department of Biomedical Sciences, Texas A&M Health Science Center, Baylor College of Dentistry, 3302 Gaston Avenue, Dallas, TX 75246, USA

## Abstract

The purpose of this retrospective study was to compare the outcome of primary endodontic treatment using a standardized cleaning and shaping technique and obturation with either lateral compaction or carrier-based obturation. Patients received primary endodontic treatment in the predoctoral dental clinic using a standardized cleaning and shaping protocol. All root canals were obturated using AH Plus^TM^ sealer with lateral compaction of gutta-percha (LC) or carrier-based obturation (CBO). A total of 205 cases met the inclusion criteria. 71 teeth in 60 patients were recalled after 2 years and evaluated both clinically and radiographically by two independent examiners. Success was defined as a lack of clinical symptoms and a normal periodontal ligament space or reduction in size of a previously existing periapical radiolucency. Chi-square and logistic regression were used for statistical analysis with a significance level of *P* < 0.05. There was no difference in success rates between cases obturated with LC or CBO (*P* = 0.802); overall success rate was 83%. Molars had a significantly lower success rate (53%) than premolar and anterior teeth (89%) (*P* = 0.005), irrespective of the obturation technique used. When a standardized cleaning and shaping protocol was used by predoctoral dental students in a controlled university setting, there was no difference in success rates between cases obturated with LC or CBO.

## 1. Introduction

The goal of root canal treatment (RCT) is the prevention and treatment of apical periodontitis. Apical periodontitis is the direct result of bacterial contamination of the root canal system and the subsequent immune response of the surrounding periapical tissues [1, 2]. During RCT the root canal system is accessed and the canals are shaped using endodontic files to remove vital tissue or necrotic debris and to facilitate irrigation and disinfection. After thorough disinfection the canal system is then obturated. The primary objective of obturation in RCT is to prevent communication of bacteria from the oral cavity through the root canal system and into the periapical tissues. Additionally, obturation prevents the ingress of apical fluids and prevents the growth of any residual bacteria left in the canal system. Complete filling of the root canal system using a semisolid core such as gutta-percha (GP) and sealer is critical in accomplishing these goals [3]. An inadequate seal can result in contamination of the canal system and can lead to periapical disease [4]. There have been a variety of techniques developed to achieve a complete filling of the root canal system including lateral compaction (LC), warm vertical compaction (WVC), and carrier-based obturation (CBO).

Lateral compaction of GP is the most commonly taught technique in dental schools in the United States [5, 6]. It has long been used as the gold standard in comparison to more newly developed techniques; however, many of these studies have been performed in vitro [7–9]. LC involves fitting a standard master cone of GP matching the last file used. Sealer is applied, the master cone is seated, and a tapered spreader is vertically placed to compact the GP laterally, providing space for additional accessory gutta-percha cones. The process is repeated until the canal is completely filled. The technique is relatively simple and cost-effective; however, it may not adequately fill canal irregularities as well as other techniques [9].

Carrier-based obturation was first described in 1978 and involved the coating of endodontic files with thermoplasticized GP [10]. One contemporary carrier-based system, Thermafil (TF; Tulsa Dental, Tulsa, OK), uses specialized plastic carriers coated with GP that are thermoplasticized in a special oven prior to insertion into the canal. The technique has been studied using in vitro models which have resulted in either no statistically significant difference or significantly better performance than LC with respect to sealing ability and filling of canal irregularities [11–15]. Following cleaning and shaping, this technique involves placing a size verifier that will correspond to the correct size obturator to be used. The canal walls are then lightly coated with sealer and a heated TF obturator is inserted with firm but passive pressure. The plastic carrier is subsequently severed at the canal orifice leaving the plastic carrier and GP as the permanent filling. The advantage of this technique is the use of a carrier to compact thermoplasticized GP and sealer both laterally and vertically more rapidly than other techniques [11].

 There have been many studies comparing obturation methods in vitro but very few in a supervised clinical setting. One prospective clinical study compared LC and WVC and found that the latter had a higher success rate only in teeth with preoperative periapical lesions [16]. The “Toronto Study” also reported higher success rates for WVC compared to LC; however, this study did not utilize a standardized cleaning and shaping protocol [17]. Another prospective clinical study found no difference in success rates when obturating with Soft-Core (CMS-Dental Aps, Copenhagen, Denmark) or LC [18]. Soft-Core is another CBO method that is similar in design and technique to TF. A Medline search revealed that only one clinical comparison of LC and TF existed in the endodontic literature [19]. The study did not find any difference in clinical outcomes between the LC and TF groups. Unlike the current study the operators performed endodontic treatment with only stainless steel hand files and had confounding variables such as the use of calcium hydroxide paste and Ledermix (Lederle Pharmaceuticals, Cyanamid GmbH, Wolfratshausen, Germany) as interappointment dressings. It is important to obtain more long-term clinical evidence comparing the outcomes of various obturation systems. The aim of the current study is to provide a direct clinical comparison of two obturation methods using standardized clinical protocols performed by undergraduate dental students under direct supervision of endodontic faculty.

## 2. Materials and Methods

This retrospective clinical study involved the evaluation of patients who received primary RCT at the Texas A&M Health Science Center/Baylor College of Dentistry predoctoral dental clinic from June 2008 to May 2009. Patients were invited to participate in the study if they met the following criteria: age 18 to 65, generally healthy (ASA I or II), and the treated tooth had been restored with a permanent restoration or full-coverage crown with or without a post [20]. The recall time ranged from 18–37 months (average 28 months). The exclusion criteria included pregnant women, any subsequent endodontic procedures performed on the tooth being investigated (e.g., endodontic retreatment, apical surgery, etc.), and severe periodontal disease. Mail contact was made to all patients who met the qualifying criteria.

 All patients had RCT under direct supervision of an endodontist in the predoctoral clinic. Predoctoral students completed a semester of didactic and hands on laboratory course work with either LC or CBO in extracted teeth. All treatment followed a standard protocol: rubber dam isolation, working length radiographs, and canals prepared using a crown-down technique with ProFile rotary instruments (DENTSPLY Tulsa Dental, Tulsa, OK, USA). Irrigation was performed between each file with 3% sodium hypochlorite, using at least 10 mL throughout the procedure. EDTA-containing paste (RC-Prep; Premier Dental Products, Philadelphia, PA, USA) was used to aid in negotiation of canals when needed. Final working length was verified radiographically with a GP master cone for the LC group or a size verifier file for the CBO group. In both groups, AH Plus (DENTSPLY DeTrey GmbH, Konstanz, Germany) was placed into the canals with a paper point. Calcium hydroxide paste (UltraCal XS, Ultradent Products Inc., South Jordan UT, USA) was placed in the canals if the case was not completed in a single visit. Obturation was performed as previously described, following the manufacturer's protocol for the CBO group. All radiographs were acquired with intraoral digitized phosphor storage plates using a paralleling technique with a film holder. Teeth were subsequently restored with a permanent restoration defined as an intracoronal restoration (amalgam or resin-composite) or a full-coverage crown.

Patient contact information was obtained from the electronic database containing the names of all patients receiving RCT during the study period. Patients were contacted by mail to join the study. Two examiners (R. Hale, R. Gatti) performed clinical and radiographic recall examinations on the patients who accepted the invitation to participate in the study. The study protocol was approved by the institutional IRB and informed consent was obtained from each patient.

### 2.1. Clinical Examination

Palpation and percussion tests were performed and patient responses were recorded. Mobility and periodontal probing depths were recorded as well as the presence of soft tissue pathosis such as a sinus tract. Clinical success was defined as no palpation or percussion tenderness with normal mobility and no soft tissue pathosis.

### 2.2. Radiographic Examination

Digitized preoperative radiographs were attained from the patients' electronic records. Postoperative digital periapical radiographs were taken using a paralleling technique with a film holder and a digital sensor (Schick Technologies, Inc., Long Island City, NY, USA). Radiographs were compared on a 23-inch LCD high-definition (1920 × 1080 resolutions) computer monitor (Asus, Taipei, Taiwan) in low light conditions. Each follow-up radiograph was analyzed for length of fill, voids, and periapical status. Length of fill was classified into groups of “acceptable” (0–2 mm from radiographic apex), “short” (>2 mm from radiographic apex), or “long” (beyond radiographic apex). Voids were classified depending on their location within the root canal system (none, coronal third, middle third, apical third). If space was present between a post and obturation material, it was included as a void. For simplicity, if teeth with multiple canals had voids in more than one canal, the most apical void was the location recorded for that tooth. Periapical status was recorded based on comparisons with preoperative radiographs and classified as one of the following: healed (normal or slightly widened PDL), healing (reduction in size of periapical radiolucency (PARL), or nonhealing (PARL unchanged, increasing in size or new PARL) [21, 22]. Radiographic success was defined as classifications of “healed” or “healing” according to the AAE definitions for measuring outcomes [23]. Radiographic failure was defined as classification of “non-healing.”

Examiners additionally recorded number of canals, initial pulpal and periapical diagnoses, days between initiation and completion of the root canal treatment, days from obturation to permanent restoration, and presence of a post or full-coverage crown.

Overall treatment success was defined as both radiographic and clinical success. Overall treatment failure was defined as radiographic failure or clinical failure. Data were analyzed using SPSS 17 (SPSS Inc., Chicago, IL, USA). Statistical analysis was performed using chi-square, Mann-Whitney, and logistic regression analysis. All tests were interpreted at the 5% significance level.

## 3. Results

A total of 71 teeth in 60 patients were included in this study. The patients were 20–66 years of age (mean =  45 ± 12  years). Among the teeth recalled, 35 received CBO and 36 received LC as root canal fillings (Table 1). The median recall time was 28 months (range 18–37 months). Of the teeth recalled, none had been extracted. The interexaminer agreement of preoperative and postoperative radiographic analysis was 100%. Power analysis of a sample size of 71 with an estimated effect size of 20% yields a power of 0.37.

A significant difference in distribution between CBO and LC groups was present with respect to recall period (*P* = .002), tooth type (*P* = .017), presence of extracoronal restoration (*P* = .008), and presence of a post (*P* = .008) (Table 1). There was no difference between groups with respect to patient age, interappointment days, time to restoration, preoperative pulp vitality, or presence of preoperative apical periodontitis.

A total of 6 teeth were classified as failures in the CBO group. Of these, 3 were classified as clinical failures, 3 were classified as radiographic failures, and none were classified as combined clinical and radiographic failures, resulting in an 83% success rate (Table 2). A total of 7 teeth were classified as failures in the LC group. Of these, 3 were classified as clinical failures, 1 was classified as a radiographic failure, and 3 were classified as combined clinical and radiographic failures, resulting in an 81% success rate.

According to the chi-square analysis, there was no significant difference in the clinical, radiographic, or treatment success between the CBO and LC groups (*P* > .05) (Table 2). Presence of extracoronal restoration, presence of post, length of obturation, presence of voids, sex, age, recall interval, and interappointment time were also found to have no statistically significant influence on treatment outcome (Table 3). Preoperative pulpal status, preoperative apical periodontitis, and days to restoration were not statistically significant but suggested a possible trend towards statistical significance (*P* = 0.080, *P* = 0.077, and *P* = 0.088 resp.). Tooth type and number of canals were the only variables found to have a significant effect on outcome (*P* = 0.005 and *P* = 0.049, resp.).

Since tooth type was found to have a significant impact on treatment outcome, the teeth were stratified by type and an additional chi-square analysis was performed. No significant difference in treatment outcome between CBO and LC groups was found for any tooth type (Table 4). There was no difference in the length of obturation between CBO and LC groups (Table 5); however, the presence of voids was statistically higher in the LC group (*P* = 0.017).

## 4. Discussion

The absence of clinical signs and symptoms of pain and swelling and radiographic appearance of normal periapical tissues have been the criteria used to assess endodontic treatment outcomes [24]. The absence of pain and swelling is a well-accepted indication of success [25]. Radiographic interpretation can be much more subjective but is still an important aspect of determining the health of an endodontically treated tooth. Several studies have shown that radiographs alone are inadequate to determine success of root canal treatment [26–29]. Clinical symptoms may indicate that there is existing posttreatment disease that cannot be depicted on a two-dimensional radiographic image. Conversely, asymptomatic teeth may be found to have persistent periapical anomalies when observed radiographically. Thus, the collection of both clinical and radiographic data is essential to evaluate treatment outcomes. Several recent studies have defined successful treatment as the absence of clinical symptoms in conjunction with a normal periodontal ligament space or a reduction in size of a previously existing radiolucency [21, 24, 30].

Using these criteria, there was no difference in the overall success rate of the LC and CBO groups in this study. This supports the findings of Chu et al. [19]. Two other prospective clinical studies directly compared LC to other obturation methods. No difference in overall success rates was found between LC and Soft-Core or LC and WVC [16, 18]. In vitro studies are mixed as to the superiority of CBO techniques over LC with respect to sealing ability. While this study did not evaluate seal, the results parallel several laboratory experiments showing that LC and CBO produce a similar seal [12, 31]. In turn, these results suggest that when adequate cleaning and shaping protocols are used, properly performed obturation techniques have minimal to no effect on clinical outcomes [12, 16, 18, 19, 31].

The overall success rate of RCT performed by predoctoral dental students in this study was 82%. A recent review article reported success rates ranging from 68 to 85% when including studies with at least a one-year recall and a strict definition of success [25]. Ng et al. also reported the results of 10 studies in which the operators were predoctoral dental students. The weighted success rate for these studies was 74.8% (range 67.0–82.7%). The current study appears to be in the upper range of reported success rates for predoctoral dental students. This could be explained by a high percentage of cases in this study with vital pulps and normal periapical tissues preoperatively as well as the direct supervision by endodontists.

Tooth type and number of canals had a statistically significant effect on treatment outcomes. Molars had a much higher failure rate than anterior and premolar teeth. It is suspected that molars add considerable difficulty to root canal treatment especially for dental students with limited clinical experience. This is in agreement with several studies reporting tooth type as a prognostic factor for root canal treatment [32–34].

Several other factors are thought to influence the outcome of endodontic treatment. These include preoperative pulp status, presence of apical periodontitis, and quality of the coronal restoration [21, 22, 30, 35, 36]. In this study, preoperative pulp status, preoperative apical periodontitis, and days to restoration did not demonstrate significant differences but did show trends towards significance. The lack of significant differences is likely due to the low percentage of necrotic teeth and teeth with preoperative apical periodontitis in addition to a relatively small sample size. Most studies in the endodontic literature (including the current study) evaluate prognostic factors in terms of success/failure. This is in contrast to studies that evaluate prognostic factors in terms of survival. Studies looking at survival do not distinguish between cases that have radiographic or clinical success, but only whether the tooth remains in the mouth at the time of recall [37–39]. A recent study by Ng et al. found that different factors may affect survival rates, including cuspal coverage, presence of proximal contacts, serving as an abutment tooth, type of tooth, and presence of preoperative pain [37].

 One of the most difficult aspects of any outcomes assessment is the acquisition of a sufficient number of patients. Power analysis of a sample size of 71 with an estimated effect size of 20% yields a power of only 0.37. This means that statistically there is a 63% chance of concluding that there is no statistically significant difference between the groups when a difference truly exists. A common goal in clinical studies is to reach a power of 0.80, leaving only a 20% chance of making such an error. In order to reach a power of 0.80, a sample size of 186 would be necessary. In this particular study, that would be nearly equivalent to the total number of available subjects, requiring a recall of 90% of patients treated which is hardly achievable. Another weakness related to sample size is group equivalency. Table 1 shows that the LC and CBO groups had statistical differences in recall period, tooth type, presence of full-coverage crowns, and presence of posts. However, Table 3 shows that, of these, only tooth type had a statistically significant effect on outcome. When the groups were stratified by tooth type, no significant difference was found in the success rate of LC and CBO. Therefore, the differences in groups listed above likely had minimal to no impact on the outcome of this study.

 LC and CBO groups showed a significant difference in the presence of voids. Apical voids in the LC group are hypothesized to be due to a lack of deep spreader penetration after master cone placement, thus prohibiting accessory cones from reaching the apical 1–3 mm. Allison et al. demonstrated that in vitro apical dye leakage correlated to the apical extent of the spreader penetration when obturating with LC [40]. Several factors seem to affect spreader penetration. Nickel-titanium spreaders are more effective than stainless steel spreaders, and the use of  .02 taper GP cones is more effective than greater taper cones [41]. When voids were present in the CBO group, they were almost always related to a gap between a post and obturation material. This is likely due to improper post fitting and cementation techniques. The combination of thermoplasticized GP and a plastic carrier acting as a compactor inserted close to working length seems to minimize the presence of voids when compared to lateral compaction. One of the critiques of any CBO technique is the risk of extruding sealer and GP from the apical foramen, although there are conflicting results in the literature. Levitan et al. suggested that the length of fill may be difficult to control using TF and is dependent on the rate of insertion [42]. Several studies have found CBO to have a statistically higher incidence of sealer extrusion than LC in an in vitro setting [43–45]. However, Abarca et al. found no difference in the amount of sealer extrusion between CBO and LC in a similar experiment [46]. The current study found no difference in length of fill between groups, suggesting that even inexperienced operators can produce consistent fills with CBO when proper shaping protocols are followed and there is an understanding of the nuances of an obturation system.

 Another critique of CBO is the possibility of the plastic carrier being stripped of GP, especially in the apical third, allowing the carrier to be in direct contact with the canal walls. One study was able to demonstrate this phenomenon by obturating and then serial sectioning curved plastic blocks [47]. However, this study utilized the older Thermafil system which used metal carriers which were less flexible than the current plastic-based system. More recent studies seem to refute these findings, suggesting that CBO has a higher percentage of the apical third filled with GP than LC [48]. It has also been suggested that cases in which the carrier becomes stripped are the result of improper shaping, namely, underinstrumenting, in the apical third [49].

 In the review article by Wu et al., the limitations of studies that evaluated the outcome of root canal therapy were identified [50]. One major criticism was the use of periapical radiographs for the determination of success. Normal periodontal ligament space or reduced lesion size is often used as a criterion for healing. However, De Paula-Silva et al. reported that 80% of cases that appeared to be healing based on periapical radiographs in dogs actually showed an increase in size when analyzed by cone-beam computed tomography (CBCT) [51]. Future studies should attempt to use CBCT technology in determining outcomes.

 In summary, within the parameters of this study, there was no difference in success rate when comparing obturation with LC or CBO performed by dental students in a controlled university setting. Tooth type significantly affected outcome, with molars having lower success rates, irrespective of obturation technique.

## Figures and Tables

**Table 1:** Distribution of independent variables among groups.

	CBO (*n* = 35)	LC (*n* = 36)	
Patient age (year)	42 (± 11)	47 (±13)	NS
Recall period (days)	800 (±136)	896 (±119)	*P* = .002
Interappointment (days)	15 (±21)	26 (±38)	NS
Time to restoration (days)	58 (±64)	60 (±76)	NS
Tooth type			*P* = .017
Anterior	21	10	
Premolar	10	15	
Molar	4	11	
Preop pulp status			NS
Vital	26	26	
Necrotic	9	10	
Preop apical periodontitis			NS
Yes	4	6	
No	31	30	
Full-coverage crown			*P* = .008
Yes	19	30	
No	16	6	
Post			*P* = .008
Yes	11	21	
No	24	15	

NS: not statistically significant (*P* > .05).

**Table 2:** Clinical and radiographic status of treated teeth at recall.

	CBO (*n* = 35)	LC (*n* = 36)	
	*n* (%)	*n* (%)	
Success			
No clinical or			
radiographic failure	29 (83)	29 (81)	NS
Failure			
Clinical failure	3 (9)	4 (11)	NS
Radiographic failure	3 (9)	6 (17)	NS
Combined clinical and			
Radiographic failure	0 (0)	1 (3)	NS

NS: not statistically significant (*P* > .05).

**Table 3:** The effects of treatment variables on outcome.

	Treatment outcome (%)		
	Success	Failure	
Obturation technique			NS
CBO	29 (83)	6 (17)	
Lateral compaction	29 (81)	7 (19)	
Tooth type			*P* = 0.005
Anterior	27 (87)	4 (13)	
Premolar	23 (92)	2 (8)	
Molar	8 (53)	7 (47)	
Number of canals			*P* = 0.049
Single	43 (88)	6 (12)	
Multiple	15 (68)	7 (32)	
Preop pulp status			NS (*P* = 0.08)
Vital	45 (87)	7 (13)	
Necrotic	13 (68)	6 (32)	
Preop apical periodontitis			NS (*P* = 0.077)
Yes	9 (69)	4 (31)	
No	52 (90)	6 (10)	
Restoration			NS
Extracoronal	38 (78)	11 (22)	
Intracoronal	20 (91)	2 (9)	
Post			NS
Yes	28 (88)	4 (12)	
No	30 (77)	9 (23)	
Length of obturation			NS
Acceptable (0–2 mm from apex)	48 (83)	10 (17)	
Long (beyond apex)	10 (83)	2 (17)	
Short (>2 mm from apex)	0 (0)	1 (100)	
Presence of voids			NS
Apical	7 (70)	3 (30)	
Middle	16 (89)	2 (11)	
Coronal	1 (50)	1 (50)	
None	34 (83)	7 (17)	
Sex			NS
Male	15 (71)	6 (29)	
Female	43 (86)	7 (14)	
Age (median)	45.5	45	NS
Recall interval (median)	837	858	NS
Interappointment time (median)	7.5	11	NS
Days to restoration (median)	20.5	72	NS (*P* = 0.088)

NS: not statistically significant (*P* > .05).

**Table 4:** Treatment success of anterior and premolar or molar teeth.

	CBO (*n* = 35)	LC (*n* = 36)	
	*n* (%)	*n* (%)	
Anterior or premolar	26/31 (83)	24/25 (96)	NS
Molar	3/4 (75)	5/11 (45)	NS

NS: not statistically significant (*P* > .05).

**Table 5:** Length of obturation and presence of voids among treatment groups.

	CBO (*n* = 35)	LC (*n* = 36)	
	*n* (%)	*n* (%)	
Length of obturation			NS
Acceptable (0–2 mm from apex)	29 (83)	29 (81)	
Long (beyond apex)	6 (17)	6 (17)	
Short (>2 mm from apex)	0 (0)	1 (3)	
Presence of voids			*P* = 0.017
Apical	1 (3)	9 (25)	
Middle*	11 (31)	7 (19)	
Coronal	1 (3)	1 (3)	
None	26 (74)	15 (42)	

*Includes voids between post and obturation material.

NS: not statistically significant (*P* > .05).
